# GABA_A_ Receptor Autoantibodies Decrease GABAergic Synaptic Transmission in the Hippocampal CA3 Network

**DOI:** 10.3390/ijms23073707

**Published:** 2022-03-28

**Authors:** Amélie F. Menke, Fatme Seval Ismail, Klaus Dornmair, Manuela Cerina, Sven G. Meuth, Nico Melzer

**Affiliations:** 1 Department of Neurology, Institute of Translational Neurology, University of Münster, 48149 Münster, Germany; a_menk04@uni-muenster.de (A.F.M.); manuela.cerina@gmail.com (M.C.); 2 Department of Neurology, University Hospital Knappschaftskrankenhaus Bochum, Ruhr University Bochum, 44892 Bochum, Germany; fatmeseval.ismail@kk-bochum.de; 3 Institute of Clinical Neuroimmunology, Biomedical Center and Hospital of the Ludwig-Maximilians-Universität München, 82152 Martinsried, Germany; klaus.dornmair@med.uni-muenchen.de; 4 Munich Cluster for Systems Neurology (SyNergy), Ludwig-Maximilians-Universität München, 81377 München, Germany; 5 Department of Neurology, Heinrich-Heine University of Düsseldorf, 40225 Düsseldorf, Germany; svenguenther.meuth@med.uni-duesseldorf.de

**Keywords:** GABA_A_ receptor autoantibody, hippocampal CA1/CA3, electrophysiology

## Abstract

Autoimmune encephalitis associated with antibodies (Abs) against α1, β3, and γ2 subunits of γ-aminobutyric acid receptor A (GABA_A_R) represents a severe form of encephalitis with refractory seizures and status epilepticus. Reduction in inhibitory GABAergic synaptic activity is linked to dysfunction of neuronal networks, hyperexcitability, and seizures. The aim in this study was to investigate the direct pathogenic effect of a recombinant GABA_A_R autoantibody (rAb-IP2), derived from the cerebrospinal fluid (CSF) of a patient with autoimmune GABA_A_R encephalitis, on hippocampal CA1 and CA3 networks. Acute brain slices from C57BL/6 mice were incubated with rAb-IP2. The spontaneous synaptic GABAergic transmission was measured using electrophysiological recordings in voltage-clamp mode. The GABA_A_R autoantibody rAb-IP2 reduced inhibitory postsynaptic signaling in the hippocampal CA1 pyramidal neurons with regard to the number of spontaneous inhibitory postsynaptic currents (sIPSCs) but did not affect their amplitude. In the hippocampal CA3 network, decreased number and amplitude of sIPSCs were detected, leading to decreased GABAergic synaptic transmission. Immunohistochemical staining confirmed the rAb-IP2 bound to hippocampal tissue. These findings suggest that GABA_A_R autoantibodies exert direct functional effects on both hippocampal CA1 and CA3 pyramidal neurons and play a crucial role in seizure generation in GABA_A_R autoimmune encephalitis.

## 1. Introduction

The γ-aminobutyric acid receptor A (GABA_A_R) is a pentameric ligand-gated chloride channel consisting of different subunits (α1–6, β1–3, γ1–3, δ, ε, π, θ, and σ1–3). GABA_A_R mediates phasic synaptic and tonic extrasynaptic inhibition [1,2,3]. Epilepsy and epileptic encephalopathies, as well as other neuropsychiatric disorders such as anxiety disorders, schizophrenia, and depression, have been related to dysfunctional GABA_A_R, e.g., by mutations within α1 or β3 subunits [1,4]. Moreover, a severe form of autoimmune encephalitis with refractory seizures, status epilepticus, and antibodies (Abs) against α1, β3, and γ2 subunits of GABA_A_R has been reported as a new form of central nervous system (CNS) autoimmunity [5,6,7,8]. Several studies have demonstrated the pathogenic effects of these antibodies on GABA_A_R function [5,9,10,11,12,13]. These Abs led to the reduction in synaptic GABA_A_R complexes, presumably via cross-linking and the subsequent internalization of the Ab-receptor complex [5,9,10]. This caused selective reduction in the postsynaptic GABA_A_R clusters at inhibitory GABAergic synapses that was linked to the hyperexcitability and dysfunction of neuronal networks [5,9,10]. In contrast, a recent study showed that recombinant human monoclonal Abs (mAbs) with GABA_A_R reactivity, which were derived from a patient with GABA_A_R encephalitis and generated using single-cell cloning, reduced inhibitory postsynaptic signaling in neuronal cultures without causing receptor internalization [12]. Subsequently, a severe clinical phenotype with epileptic seizures was induced by cerebroventricular infusion of GABA_A_R mAbs into rodents [12]. In another study, a recombinantly expressed pathogenic antibody from the cerebrospinal fluid (CSF) B cells of a patient with autoimmune GABA_A_R encephalitis, previously described and termed “index patient 2” (IP2), led to the reduction in phasic GABAergic inhibitory synaptic activity and the increase in excitability in hippocampal CA1 pyramidal neurons, most probably contributing to clinical disease symptoms [13]. The CA1 pyramidal cells of the hippocampus received input from other hippocampal fields such as CA3 [14]. There is evidence that the epileptiform-discharges-like sharp waves are correlated with the synchronous discharges of pyramidal cells in CA1 and CA3 fields, of dentate granule cells, and of interneurons [15]. In mesial temporal lobe epilepsy, it has been supposed that interictal spikes are initiated in the CA3 field of the hippocampus by pacemaker pyramidal cells and propagated as population bursts throughout the CA3 subfield to the CA1 subfield via the Schaffer collaterals [15,16,17,18,19]. The CA1 subfield is responsible for further propagation and spread of the interictal spikes to the subcortical brain structures outside the hippocampus via the subiculum and the entorhinal cortex [15,20].

Studies of the influence of GABA_A_R Ab on signal transduction in hippocampal CA3 pyramidal neurons are missing. Therefore, our aim in this study was to investigate the direct pathogenic effect of the recombinant GABA_A_R antibody (rAb-IP2), derived from the CSF of a patient with GABA_A_R encephalitis, on the hippocampal CA3 network in addition to CA1.

## 2. Results

### 2.1. GABA_A_ Receptor Autoantibodies (rAb-IP2) Reduce Inhibitory Postsynaptic Signaling in Hippocampal CA1 Pyramidal Cells

**Figure 1:**
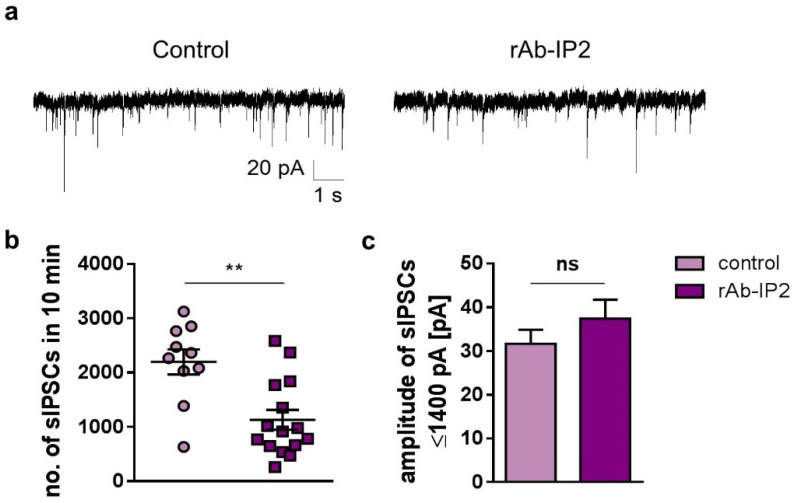
The recombinant GABA_A_ receptor autoantibody rAb-IP2 reduced the number of spontaneous inhibitory postsynaptic currents (sIPSCs) generated in CA1 pyramidal neurons. (**a**) Exemplary traces depict the GABAergic synaptic transmission in rAb-IP2-incubated pyramidal neurons and controls. Notably, the exemplary traces are cropped at 600 pA to simplify illustration and do not represent the full high-amplitude currents with an amplitude > 1400 pA, which were very likely contaminated with Na^+^ currents and were not GABA-mediated. (**b**) Scatter plot showing that the number of sIPSCs recorded in 10 min decreased upon rAb-IP2 incubation in comparison with the control. (**c**) Bar graphs showing the amplitude of sIPSCs in rAb-IP2-incubated neurons and controls. The amplitude of sIPSCs ≤ 1400 pA was not altered by rAb-IP2 incubation. ** *p* < 0.01, ns: not significant.

To characterize the effect of the recombinant antibody rAb-IP2 on GABA_A_R function in different hippocampal regions, we performed electrophysiological experiments using acute murine brain slices. The GABAergic activity of pyramidal neurons was recorded in hippocampal CA1 and CA3, in which the α1 and β3 subunits are frequently expressed on GABA_A_Rs [21,22]. In CA1, the incubation of brain slices with rAb-IP2 for 2 h led to a significant decrease in the number of spontaneous inhibitory postsynaptic currents (sIPSCs) recorded in a period of 10 min, compared with the control group without antibody application during the incubation period (rAb-IP2: 1130 ± 181.8, *n* = 10; control: 2198 ± 232.7, *n* = 15; unpaired Student’s *t*-test: *t* = 3.65, df = 23, ** *p* = 0.001; Figure 1a,b). The amplitude of GABAergic-mediated currents was not altered upon rAb-IP2 incubation, compared with the control (rAb-IP2: 59.71 ± 10.05 pA; control: 127.8 ± 42.89 pA; unpaired Student’s *t*-test: *t* = 1.85, df = 23, *p* = 0.08). Remarkably, high-amplitude ion currents were present during recordings in CA1 and CA3 pyramidal neurons, which were previously not commonly reported in these hippocampal areas. The currents were measured to have an amplitude > 1400 pA in contrast with sIPSCs with an amplitude ≤ 1400 pA. These high-amplitude currents were very likely contaminated with Na^+^ currents; therefore, they were not considered in the following steps. After excluding these recorded high-amplitude currents with an amplitude > 1400 pA which were not GABA-mediated, it appeared that the amplitude of sIPSCs < 1400 pA was not reduced by rAb-IP2 incubation (rAb-IP2: 37.39 ± 4.35 pA; control: 31.67 ± 3.21 pA; unpaired Student’s *t*-test: *t* = 0.96; df = 23, *p* = 0.35; Figure 1c). In summary, rAb-IP2 decreased GABAergic signaling in CA1 pyramidal neurons with regard to the number of sIPSCs, but not with regard to the amplitude.

### 2.2. GABA_A_ Receptor Autoantibodies Decrease GABAergic Synaptic Transmission in Hippocampal CA3 Network

**Figure 2:**
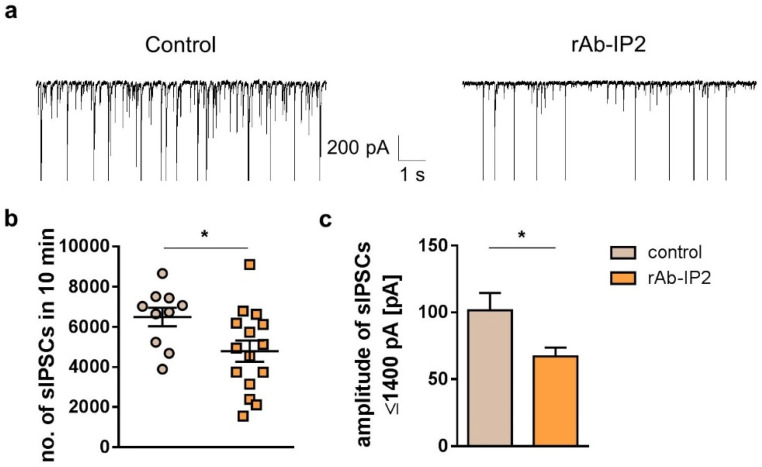
Incubation with the recombinant GABA_A_ receptor autoantibody rAb-IP2 led to both decreased number and amplitude of spontaneous inhibitory postsynaptic currents (sIPSCs) in pyramidal CA3 neurons. (**a**) Exemplary traces show GABA-mediated synaptic currents in rAb-IP2-incubated pyramidal neurons and controls. For illustration purposes, the amplitude of the high-amplitude currents is cut at 800 pA. (**b**) rAb-IP2-incubated neurons showed a significantly reduced number of sIPSCs in comparison with controls. (**c**) rAb-IP2 led to a significant reduction in the amplitude of sIPSCs when sIPSCs ≤ 1400 pA were analyzed. * *p* < 0.05, ns: not significant.

In the next step, the results of CA1 were compared with those of the CA3 hippocampal region, which plays a critical role in the initiation of epileptic activity. The CA3 pyramidal neurons showed a significantly reduced number of sIPSCs upon rAb-IP2 incubation (rAb-IP2: 4788 ± 530.5, *n* = 10; control: 6487 ± 458.2, *n* = 15; unpaired Student’s *t*-test: *t* = 2.259, df = 23, * *p* = 0.03; Figure 2a,b). The amplitude of sIPSCs tended to be reduced after rAb-IP2 incubation (rAb-IP2: 227.7 ± 40.68 pA; control: 374.9 ± 73.89 pA, *n* = 11; unpaired Student’s *t*-test: *t* = 1.867, df = 24, *p* = 0.07, *n* = 15). Focusing on sIPSCs ≤ 1400 pA, the amplitude of GABAergic postsynaptic currents was significantly reduced after rAb-IP2 incubation (rAb-IP2: 67.14 ± 6.51 pA; control: 101.6 ± 12.94 pA; unpaired Student’s *t*-test: *t* = 2.577, df = 24, * *p* = 0.02; Figure 2c). In summary, the recombinant antibody rAb-IP2 led to both decreased number and amplitude of sIPSCs in CA3 pyramidal neurons.

### 2.3. Immunohistochemical Staining Confirmed GABA_A_ Receptor Autoantibody Binding to Hippocampal Tissue

**Figure 3:**
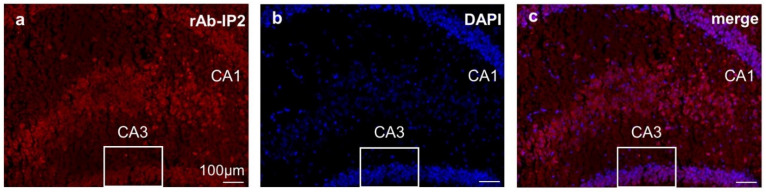
Immunohistochemical staining of a formalin-fixed, paraffin-embedded murine hippocampus with the recombinant human GABA_A_ receptor autoantibody rAb-IP2. Murine-fixed hippocampal tissue shows immunoreactivity to the recombinant human antibody rAb-IP2. (**a**) Representative coronal hippocampal slices with immunoreactivity to rAb-IP2 (red). The box marks the hippocampal region CA3, showing the area used for the in vitro electrophysiological recordings as shown in Figure 1 and Figure 2. For more visibility of CA1, we also referred to our previous study [13]. (**b**) DAPI (blue) was used as a marker for the cell nuclei. (**c**) Merged picture. Scale bars represent 100 μm.

The hippocampal slices showed strong immunoreactivity to rAb-IP2 compared with the negative controls (Figure 3a–c). The staining confirmed that the human autoantibody cross-reacts with rodent brain structures.

## 3. Discussion

In this study, the recombinant GABA_A_ receptor autoantibody (rAb-IP2) reduced inhibitory postsynaptic signaling in hippocampal CA1 and CA3 pyramidal cells. Our results confirmed previous findings showing rAb-IP2-induced reduction in spontaneous postsynaptic GABAergic events in hippocampal CA1 pyramidal neurons [13]. Furthermore, this was linked to increased excitability in hippocampal CA1 pyramidal neurons. All these alterations in GABAergic synaptic transmission were assumed to cause the clinical symptoms of patients with GABA_A_R encephalitis [5,7,8]. Additionally, in our study, the GABAergic synaptic transmission was also decreased in CA3 pyramidal neurons. This finding is important because CA3 neurons are involved in the generation of epileptic discharges [15,16,17,18,19]. In another study, the cerebroventricular infusion of GABA_A_R mAb in Wistar rats caused spontaneous seizures in vivo [12]. Moreover, increased spontaneous epileptic activity was detected from electrodes placed in the hippocampal areas CA1 and CA3 of the rats ex vivo [12]. In line with these findings, incubation of cultured autaptic neurons with GABA_A_R mAb reduced inhibitory postsynaptic signaling in vitro [12]. These effects were observed independent of receptor internalization, indicating that GABA_A_R autoantibodies exert direct functional effects on the CA1 and CA3 pyramidal neurons and play a crucial role in seizure generation in GABA_A_R autoimmune encephalitis. The exact pathomechanism for Ab-mediated effects is yet not known. Multiple possible mechanisms were suggested, including receptor modulation, e.g., by desensitized conformation, redistribution, or network effects [12]. Further studies revealing the exact Ab-mediated pathomechanisms are needed. The target epitope of the mAb involved mainly the α1 and γ2 subunits [12]. In addition, in a complementary assay using flow cytometry, the mAb bound to GABA_A_Rs, coexpressing the α1, γ2, and β3 subunits [12]. It is important to consider that GABA_A_R subunit expression and composition significantly vary among different brain regions and subcellular locations [22,23,24]. This large heterogeneity of possible subunit combinations implies differences in the electrophysiological properties of the GABA_A_R [25,26]. For example, the presence of the α1 subunit is responsible for the fast decay time kinetics in IPSCs [9]. The varying expression of α1 and β3 subunits in the stratum pyramidale of CA1 and CA3 might explain why the amplitude and quantity of sIPSCs are affected in different ways by rAb-IP2. Moreover, it was shown that decreased expression of the α1-subunit mRNA correlated with altered GABA_A_R function and neuronal excitability in single dentate granule cells in an animal model of temporal lobe epilepsy [27]. The mutations in the GABA_A_R α1 and γ2 subunits have been related to different idiopathic generalized epilepsy syndromes [28,29]. These results confirmed that aberrant GABA_A_R expression and function play an essential role during epileptogenesis. Patients with GABA_A_R encephalitis develop frequent seizures, including refractory status epilepticus or epilepsia partialis continua [5,6]. Consistent with these clinical data, epileptiform activity was detected using wireless electroencephalography (EEG) in living animals that received GABA_A_R mAb as an infusion [12]. A peak of ictal events was achieved under the GABA_A_R mAb infusion, and the events persisted until 14 d after termination of the infusion [12]. In contrast, in animal models of N-methyl-D-aspartate receptor (NMDAR) encephalitis, memory deficits remained 4 d after the infusion was stopped and then resolved within the next 7 d [30]. GABA_A_R mAb led to higher EEG coastline length in the infused animals and to significantly higher power in all the power band ranges, especially in the lower frequency range (1–4 Hz), which is in line with EEG features in NMDAR encephalitis [12,31]. Similar findings with increases in the theta and delta powers were detected in the kainate-induced status epilepticus in mice [32]. A recent study demonstrated that the intracerebroventricular injection of NMDAR Abs in rats led to a higher number of interictal events in the CA3 hippocampal region compared with the CA1, and to a spontaneous epileptic activity, highest in the CA3 region [33]. Additionally, whole-cell patch-clamp recordings from hippocampal CA3 pyramidal cells after the injection of NMDAR Abs showed a reduction in excitatory, but not in inhibitory, synaptic neurotransmission, or intrinsic hyperexcitability. The reduced synaptic excitatory neurotransmission is assumed to underlay seizures in this rat model of NMDAR Ab-mediated encephalitis [33]. In our study, GABA_A_R Abs led to decreased number and amplitude of sIPSCs in CA3 pyramidal neurons, the injection of NMDAR Abs caused no changes in the frequency or amplitude of sIPSCS. Following this, each CNS autoantibody may contribute in different ways to excitatory–inhibitory imbalance, which plays a critical role in the pathophysiology of seizures. The neuronal circuits are highly complex, and changes in synaptic transmission can have extensive impacts on the stability of neuronal networks.

It can be summarized that GABA_A_R autoantibodies exert direct functional effects on the hippocampal CA1 and CA3 pyramidal neurons and play a crucial role in seizure generation in GABA_A_R autoimmune encephalitis.

## 4. Materials and Methods

### 4.1. Clinical Samples 

In our study, the recombinant GABA_A_R autoantibody (named rAb-IP2) from the CSF of patient IP2, who was suffering from anti-GABA_A_R encephalitis, was cloned, expressed, purified, and characterized according to a previous study [13]. The clinical data of patient IP2 were previously described [5,13]. It was demonstrated that rAb-IP2 specifically binds to the α1 and β3 subunit of the GABA_A_R [13]. 

This study was approved by the ethics committee of Münster, Germany (AZ 2013-682-b-S). A written informed consent was obtained prior to study conduct, according to the principles of the Declaration of Helsinki. 

### 4.2. Animals

All C57BL/6 mice were kept under pathogen-free conditions and had access to food and water ad libitum. All experiments were conducted according to German law and were approved by the local authorities (Landesamt für Natur, Umwelt und Verbraucherschutz Nordrhein-Westfalen).

Immunohistochemical studies were performed on brains obtained from 10–17-week- old mice, whereas for the electrophysiological analysis, younger mice (2–7 weeks old) were used because the GABAergic system in mice is robust and no significant age-related differences in the GABA_A_R α1, α2, α3, α5, β3, and γ2 subunit expression levels were found between the young- and old-age groups in different regions of the mouse hippocampus [34]. Moreover, one additional reason for the age difference between the mice used for the two different experiments was that our laboratory follows the 3R principles, which aim to refine and reduce the number of animals used in a given experiment.

### 4.3. Preparation of Acute Murine Brain Slices

Brains were collected from C57BL/6 mice (2–7 weeks old) after anesthesia with 4% isoflurane in O_2_ and fast decapitation. Acute brain slices were obtained by cutting 260 μm thick slices using a vibratome (Leica, Wetzlar, Germany). For investigation of the hippocampal CA1 and CA3 regions, transversal slices were collected. Slices were placed in a submersion chamber and continuously perfused with an extracellular solution (artificial CSF) containing 120 mM NaCl, 2.5 mM KCl, 1.25 mM NaH_2_PO_4_, 22 mM NaHCO_3_, 20 mM C_6_H_12_O_6_, 2 mM CaCl_2_, and 2 mM MgSO_4_ (Merck, Darmstadt, Germany), set to pH of 7.35 with carbogen. 

### 4.4. Incubation of Acute Murine Brain Slices with the GABA_A_R Autoantibody rAb-IP2

Acute brain slices were incubated with the human antibody rAb-IP2 using an incubation chamber. The bottom of the chamber was filled with extracellular solution (artificial CSF), which was kept at room temperature and purged with carbogen. After centrifugation and vortexing of a batch containing rAb-IP2, the Ab was added at a final concentration of 4.84 µg/mL. The brain slices were incubated with rAb-IP2 for 2 h. Control slices were kept under the same conditions in the absence of rAb-IP2.

### 4.5. Electrophysiological Recordings by Patch-Clamp Technique

After the incubation step, the brain slices were placed in a custom-built recording chamber, which was continuously perfused with the external solution. Glass pipettes for recording were pulled from borosilicate glass (GC150TF-10; Harvard Apparatus, Holliston, MA, USA) capillaries by using a vertical pipette puller. For the voltage-clamp mode, the pipettes were filled with a KCl-based, high-chloride intracellular solution containing 10 mM NaCl, 110 mM KCl, 11 mM EDTA, 10 mM HEPES, 1 mM MgCl_2_, 0.5 mM CaCl_2_, 15 mM phosphocreatine, 3 mM Mg-ATP, and 0.5 mM Na-GTP, set to pH 7.25 with KOH and an osmolality of 295 mOsmol/kg (Sigma-Aldrich, Schnelldorf, Germany; Merck, Darmstadt, Germany). The liquid-filled glass pipette was connected to an EPC-10 amplifier (HEKA Elektronik, Lamprecht, Germany) for generating and measuring ionic currents. The electrode resistance varied between 4 and 7 MΩ, and the series resistance was 5–15 MΩ (compensation ≥ 25%). A bright-light microscopy was used for visual identification of the pyramidal neurons in the hippocampal regions. The recordings were governed by Patchmaster software (HEKA Elektronik, Lamprecht, Germany). 

#### Voltage-Clamp Analysis

In order to analyze the effects of the rAb-IP2 on GABA_A_R function, the spontaneous synaptic GABAergic transmission was measured in voltage-clamp mode. As is well-known, inhibitory postsynaptic currents (IPSCs) are induced by release of presynaptic GABA, which binds to postsynaptic GABA_A_Rs, resulting in hyperpolarization of the postsynaptic membrane. In this experimental setup, an intracellular solution with a higher concentration of Cl^−^ (117 mM) and physiological intracellular concentrations of other ions was prepared. Following this, we investigated the cells in the range of their resting membrane potential (RMP) at −65 mV and measured outwardly directed Cl^-^ currents. To evaluate only GABA-triggered ion currents, the competitive AMPA- and kainate-receptor antagonist 6,7-dinitroquinoxaline-2,3-dione (DNQX, 10 µM) and the NMDA-receptor blocker (2R)-amino-5-phosphonopentanoate (AP5, 10 µM) were added to the extracellular solution. The GABAergic activity was recorded for 10 min. The analysis of sIPSCs was performed semiautomatically using MiniAnalysis (Synaptosoft Inc., Fort Lee, NJ, USA) and FitMaster (HEKA Elektronik, Lamprecht, Germany). The number and the amplitude of sIPSCs were used as read-outs. 

### 4.6. Immunohistochemistry

After deep anesthesia of the C57BL/6 mice (10–17 weeks old), the brains were removed, placed into a cryo-protective fixative buffer (Tissue-Tek, Sakura Finetek, Alphen aan den Rijn, The Netherlands), and frozen at −20 °C. Afterward, coronal slices of 10 µm including the hippocampal region were cut using a cryotome. The slices were fixed with 10% of paraformaldehyde (PFA) (Merck, Darmstadt, Germany) for 10 min and washed three times with phosphate-buffered saline (PBS) (Sigma-Aldrich, Schnelldorf, Germany) for 5 min. Next, a blocking solution containing 1% goat serum (PAA, Pasching, Austria), 10% bovine serum albumin (Sigma-Aldrich, Schnelldorf, Germany), and 1% Triton X-100 (Sigma-Aldrich, Schnelldorf, Germany) was applied for 2 h. Afterward, the slices were treated with the primary antibody rAb-IP2 at a concentration of 1:250 at 4 °C overnight. After washing with PBS, Cy3-conjugated goat antihuman IgG was used as a secondary antibody (emission at a wavelength of 565 nm) (Dianova, Hamburg, Germany) at a concentration of 1:300 (incubation period 2 h at room temperature). The slices were stained with 5–10 µL Fluoromount G containing 4′,6-diamidino-2-phenylindole (DAPI) (Thermo Fisher Scientific, Waltham, Massachusetts, USA) for visualization of the cell nuclei. Hippocampal slices without rAb-IP2 incubation were prepared as negative controls without the detection of any fluorescent signal. For image acquisition and conducting the analysis, an Axio Scope A1 fluorescence microscope (Carl Zeiss GmbH, Jena, Germany) was used.

### 4.7. Statistics

Grubb’s test was used for testing significant outliers in the data sets. Gaussian-distributed data sets were analyzed using Student’s *t*-test for comparisons of two groups. GraphPad Prism 6 (GraphPad Software, San Diego, CA, USA) was applied to analyze and present the data in graphs. Representative traces were generated with OriginPro 2018 software (OriginLab, Friedrichsdorf, Germany). The number of experiments was reported as the number of recordings. All results are reported as mean value ± standard error of the mean (SEM). Level of significance was set as *p* < 0.05.

## 5. Conclusions

This study confirmed previous findings that GABA_A_R autoantibodies (rAb-IP2) led to reduced inhibitory postsynaptic signaling and increased excitability of pyramidal neurons in hippocampal CA1. We demonstrated further disruption of GABAergic signaling in hippocampal CA3, which is known to be a critical region for seizure generation in mesial temporal lobe epilepsy and can be one of the crucial points in the pathophysiology of GABA_A_R encephalitis. Our results support previous study data showing that the infusion of monoclonal GABA_A_R autoantibodies in rodents caused increased spontaneous epileptic activity in the hippocampal CA1 and CA3 areas ex vivo and spontaneous seizures in vivo.

Taken together, these findings strongly indicate the pathogenic effects of GABA_A_R autoantibodies on neuronal function as dampened GABAergic transmission and increased neuronal excitability, significantly contributing to seizures and status epilepticus.

## Data Availability

The datasets used or analyzed during this study are available from the corresponding author upon reasonable request.
